# Global change-type drought-induced tree mortality: vapor pressure deficit is more important than temperature per se in causing decline in tree health

**DOI:** 10.1002/ece3.664

**Published:** 2013-07-10

**Authors:** Derek Eamus, Nicolas Boulain, James Cleverly, David D Breshears

**Affiliations:** 1 Plant Biology and Climate Change Research Cluster, University of Technology SydneyBroadway, PO Box 123, Sydney, New South Wales, 2007, Australia; 2 National Centre for Groundwater Research and Training, University of Technology SydneyBroadway, PO Box 123, Sydney, New South Wales, 2007, Australia; 3 School of Natural Resources and the Environment, University of ArizonaTucson, Arizona, 85721; 4 Department of Ecology and Evolutionary Biology, University of ArizonaTucson, Arizona, 85721

**Keywords:** Drought, ET, NPP, SPA model, tree mortality, VPD

## Abstract

Drought-induced tree mortality is occurring across all forested continents and is expected to increase worldwide during the coming century. Regional-scale forest die-off influences terrestrial albedo, carbon and water budgets, and land-surface energy partitioning. Although increased temperatures during drought are widely identified as a critical contributor to exacerbated tree mortality associated with “global-change-type drought”, corresponding changes in vapor pressure deficit (*D*) have rarely been considered explicitly and have not been disaggregated from that of temperature per se. Here, we apply a detailed mechanistic soil–plant–atmosphere model to examine the impacts of drought, increased air temperature (+2°C or +5°C), and increased vapor pressure deficit (*D*; +1 kPa or +2.5 kPa), singly and in combination, on net primary productivity (NPP) and transpiration and forest responses, especially soil moisture content, leaf water potential, and stomatal conductance. We show that increased *D* exerts a larger detrimental effect on transpiration and NPP, than increased temperature alone, with or without the imposition of a 3-month drought. Combined with drought, the effect of increased *D* on NPP was substantially larger than that of drought plus increased temperature. Thus, the number of days when NPP was zero across the 2-year simulation was 13 or 14 days in the control and increased temperature scenarios, but increased to approximately 200 days when *D* was increased. Drought alone increased the number of days of zero NPP to 88, but drought plus increased temperature did not increase the number of days. In contrast, drought and increased *D* resulted in the number of days when NPP = 0 increasing to 235 (+1 kPa) or 304 days (+2.5 kPa). We conclude that correct identification of the causes of global change-type mortality events requires explicit consideration of the influence of *D* as well as its interaction with drought and temperature.

This study disaggregates the influence of temperature and vapour pressure deficit on net primary productivity of an Australian woodland and their interactions with drought as potential causal agents in recent widespread forest mortality.

## Introduction

Extreme events such as drought are recognized as drivers of vegetation change under future climate (Overpeck and Cole 27; Smith 30). Droughts have shifted vegetation boundaries substantially, as evident in both paleo- and historical (Davis et al. 10; Allen and Breshears 2) data. Although assessing trends in extreme events as opposed to mean conditions remains difficult due to much greater limitation in the available data (Dai 9; IPCC 15), recent analyses suggest an increase in aridity and drought across much of southern Europe, Africa, most of the Americas, Australia, and Southeast Asia (Dai 9). Increased frequency, severity, and aerial extent of drought and concomitant regional-scale forest dieback are of concern because of the impact this has on terrestrial albedo, C and water budgets, and land-surface energy partitioning. Transformations of vegetation cover at catchment and regional scales, with concomitant impacts on provision of ecosystem goods and services (Breshears et al. 8) are likely to be most strongly felt in developing countries.

Although attention has recently focused on the role of extreme events such as drought as a driver of climate change impacts, the need to consider the concurrent effect of trends and extremes in climate has been identified (Jentsch et al. 16). In particular, a trend in warmer temperature is superimposed upon the distribution of current and projected future drought. Climate change is associated with increased temperatures across much of the world, especially nighttime temperatures, which are increasing faster than daytime temperatures (Easterling et al. 11; Meehl et al. 26). Given the strong temperature dependence of respiration, such changes are likely to significantly alter the C balance of forests. Under future climate change, we are likely to have “global-change-type drought” – drought accompanied by warmer temperatures – that will have pronounced biophysical and ecological effects (Breshears et al. 6, 7; Overpeck and Udall 28).

Over the past 30 years, drought and heat-related mortality have been reported on every wooded continent (Allen et al. 3). Although these events cannot be unambiguously attributed to changing climate, they raise concerns that the world's wooded ecosystems may already be responding to changing climate. Warmer temperatures concurrent with drought have been implicated as causal agents of increased rates of defoliation, mortality, and large-scale die-off events, (van Mantgem et al. 21; Breshears et al. 6). Adams et al. (1) isolated the effect of warmer temperature during drought and observed that an increase in temperature of ~4°C reduced survival time during drought in a *Pinus* species by ca. 30% (Adams et al. 1). This result, when considered concurrently with the nonlinear distribution of drought frequency versus drought duration, for which shorter droughts occur much more frequently than longer droughts, suggests large increases in tree mortality under “global-change-type drought”. However, as is commonly observed in these experiments *only* temperature was controlled and with increased temperature comes increased vapor pressure deficit. While warmer temperatures were implicated in a large die-off of *Pinus edulis* in the southwestern US (Breshears et al. 6), subsequent analysis highlighted particularly pronounced differences in vapor pressure difference (*D*) between that drought event and a previous one during the 1950s that shifted ecotones but did not trigger large-scale die-off (Weiss et al. 32).

Understanding the mechanisms underlying drought- and heat-driven tree mortality is essential for improving future predictions of vegetation change. Mechanisms driving such mortality were recently reviewed (McDowell et al. 23), and these have become a topic of intense debate (Leuzinger et al. 19; Sala et al. 29; McDowell et al. 24). Proposed non-mutually exclusive mechanisms that could drive tree mortality include hydraulic failure and carbon starvation (McDowell et al. 23) and their interaction (McDowell et al. 24); biotic agents such as pests and pathogens may act as secondary factors driving mortality.

We hypothesize that (1) short-term drought has only a small-scale impact on canopy function and this will have a larger effect on water use than C gain; (2) increased temperature alone will have only a small impact on C and water relations of a canopy; (3) increased *D* alone will exert a larger negative impact on canopy function than increased temperature alone; (4) increased temperature with short-term drought has a larger negative impact on canopy function than either stress effect applied singly; (5) increased temperature and increased *D* have a larger negative impact on canopy function than either stress applied alone; (6) increased *D* (either combined with drought or increased temperature) has the largest negative impact on canopy function; and (7) long-term reductions in NPP to values of zero can be induced by increased *D* and temperature with or without drought. Thus, the aim of this work was to disaggregate the effects of temperature per se from the effects of *D* per se on canopy behavior and function. Our approach was to use a well-developed and tested model (the soil–plant–atmosphere model, SPA; Williams et al. 36) on a tree species for which ecophysiological characteristics have been well characterized. We explicitly do not consider the influence of changes in atmospheric CO_2_ concentration because we are concerned here with a discussion of recent and near-future causes of widespread mortality, where changes in CO_2_ concentration have little impact.

## Methods

### Site description

The site modeled in this study was a remnant Cumberland Plains woodland, near Richmond, 47 km northwest of Sydney, New South Wales, Australia (33° 39′S, 150° 46′E, elevation 40 m). The vegetation is an open woodland, with an average height of 14 m, dominated by *Angophora bakeri* (E. C. Hall) (narrow-leaved apple) and *Eucalyptus sclerophylla* (Blakely) L. A. S. Johnson & Blaxell (Scribbly Gum). These two species account for about 80% of total tree basal area at the site. Soils are duplex with a sandy upper profile to 0.8 m overlying a deep (>10 m) weakly pedal orange heavy clay (Bannerman and Hazelton 4). Mean tree basal area for the site was 1.45 ± 0.35 m^2^ ha^−1^ with 85.5 ± 6.5 stems ha^−1^ and leaf area index was generally about 1.3 throughout the study period. The understorey is dominated by shrubs and grasses including *Pultenaea elliptica, Cryptandra amara,* and *Melaleuca thymifolia*. The climate is temperate, with mean maximum winter (July) and summer temperatures (January) in 2006 of 17.2 and 29.3°C, respectively. Mean annual rainfall is 729 mm and is slightly summer dominant.

### The soil–plant–atmosphere model

The SPA model simulates canopy exchanges of carbon and water at 30-minute resolution for multiple (up to 10) foliage layers. The model was used in the form as described by Zeppel et al. (40); further explanation on the subroutines of SPA may be found in Williams et al. (35, 37, 38). This model requires parameterization for soil characteristics (e.g., depth and sand/silt ratio), extensive parameterization for vegetation attributes (e.g., LAI, foliar N content, root depth distribution, minimum leaf water potential), and extensive meteorological parameterization (e.g., hourly or daily values of rainfall, solar radiation, temperature, and vapor pressure deficit). Table one provides details of all soil and plant parameter values required for the model. Meteorological data and vegetation characteristics (e.g., root depth, foliar N content) from a remnant Cumberland woodland in western NSW in Australia were used as inputs (see Whitley et al. 34 and Zeppel et al. 40 for site descriptions and parameter estimation; Table 1).

**Table 1:** Values used in SPA for this study

Parameter/Variable	Symbol	Units	Value	Source
Ambient atmospheric CO_2_ concentration	C_a_	mmol mol^−1^	374	Value from recent past
Canopy layer capacitance	C_n_		2000	Williams et al. 35
Canopy hydraulic conductivity	G_p_	mmol m^−1^ sec^−1^ MPa^−1^	3.5	Zeppel et al. 40
Layer height of soil	H	m	0.1–1.0 m depth, then 0.2–3.4 m	Site estimate
Leaf area index	LAI	m^−2^ m^2^	0.1–2.3	MODIS LAI product
Areal concentration of leaf N	N	g m^−2^ ground area	0.16–3.65	Prior et al. 111 and Ghannoum et al. 109
Proportion of total canopy N in top layer	N_top_		0.125	Zeppel et al. 40
Fine root radius	r_r_	M	0.0001	Zeppel et al. 40
Air temperature	T_a_	°C	Variable	Zeppel et al. 40
Leaf temperature	T_i_	°C	Variable	Zeppel et al. 40 and varied in scenarios
RuBP carboxylation capacity	V_cmax_	μmol g^−1^ sec^−1^	27.4074	Zeppel et al. 40
Maximum electron transport rate	J_max_	μmol g^−1^ sec^−1^	48.1481	Zeppel et al. 40
δA/δ*g*_s_ threshold for stomatal opening	Ι	%	1.0007	Williams et al. 35
Minimum sustainable leaf water potential	Ψ_lmin_	MPa	−2.5	Kelley et al. 110
Soil water potential	Ψ_s_	MPa	−0.5	Predawn leaf water potential, estimated
% soil clay content in top 10 cm		%	15.0	Zeppel et al. 40
% soil sand content in top 10 cm		%	85.0	Zeppel et al. 40
Drain check – field capacity as fraction of total porosity		Fraction	0.5	Zeppel et al. 40
Latitude		°	33° 39′ 42″	
Dimension of leaves		m^2^	0.08	Prior et al. 51
Root resistivity		MPa sec g mmol^−1^	100	Estimated
Root biomass		g	2450	Chen et al. 108
Rooting depth		m	3.4	Chen et al. 108

SPA model input values indicating the name, symbol, units, value used, and whether the data were measured or estimated for the study site.

The SPA model applies the Farquhar model of leaf-scale photosynthesis (Farquhar and von Caemmerer 12) and the Penman–Monteith equation to calculate leaf-level transpiration. These two processes are linked by a model of stomatal conductance that optimizes daily C gain per unit leaf nitrogen. The difference between soil and leaf water potential and the hydraulic conductance of the soil-to-canopy pathway determines the flux of water through the plant. Temperature and vapor pressure deficit are explicitly included in the Farquhar model of photosynthesis, the Penman–Monteith equation, the calculations for stomatal conductance, and the energy balance calculations for the canopy and soil surface. The full set of equations for the model is available in the SPA Handbook available on the SPA website at the University of Edinburgh (http://www.geos.ed.ac.uk/homes/mwilliam/spa.html).

Across a 2-year simulation period, the model was run with 15 scenarios that represented natural conditions (control) and 14 experimental manipulations of three meteorological *inputs* (*D*, temperature and drought; Table 2). Drought is defined here as the unseasonal absence of rainfall for a 3-month summer period. Changes in temperature (+2°C or +5°C) and *D* (+1.5 kPa or +2.5 kPa) were applied singly or in combination from day 1 of the first year by manipulation of the meteorological input driver file. The drought treatment was imposed in December of year 1 and January and February of year 2 (Table 2).

**Table 2:** Summary of the 15 scenarios used to determine the relative impact of temperature, drought, and vapor pressure deficit on canopy function

Simulation run	Meteorological variables adjusted
1: Control	None; 2-year measured field meteorological data used as inputs
2: Simulated drought (defined as an unseasonal absence of rainfall for the three summer months)	Rainfall excluded for the summer (December year 1 to February year 2, inclusive); all days with rain in this period were replaced with hot and dry meteorological conditions; all other days were identical to the control simulation ([1] above)
3: Increased air temperature, *D* unchanged	Air temperature was increased either by 2°C or 5°C above the observed (control) data for the entire 2-year simulation. All other input data remained unchanged.
4: *D* increased, but air temperature unchanged	*D* increased either by 1 or 2.5 kPa above the observed (control) data. All other input data remained unchanged.
5: Small increase in air temperature and *D*	Air temperature increased by 2°C and *D* increased by 1 kPa throughout the 2-year simulation. All other input data remained unchanged.
6: Larger increase in air temperature and *D*	Air temperature increased by 5°C and *D* increased by 2.5 kPa throughout the 2-year simulation. All other input data remained unchanged.
7: Drought plus increased *D*, but air temperature remained unchanged	3-month summer drought (as per [2] above) and *D* increased by either 1 kPa or 2.5 kPa
8: Drought plus increased temperature, but *D* remained unchanged	3-month summer drought (as per [2] above) and temperature increased by 2 or 5°C and *D* unchanged
9: Drought plus increased *D* and small increase in temperature	3-month summer drought (as per [2] above) and *D* increased by 1.0 or 2.5 kPa with temperature increased by 2°C
10: Drought plus increased *D* and larger increase in temperature	3-month summer drought (as per [2] above) and *D* increased by 1.0 or 2.5 kPa with temperature increased by 5°C

Note that some scenario summaries (3, 4, 7, 8, 9, and 10) include two levels of manipulation.

### Outputs of the model

Key outputs examined in this study include stomatal conductance (*g*_s_), soil moisture content, and daily NPP, transpiration, and minimum leaf water potential. To evaluate treatment effects, transpiration and NPP outputs from each manipulation scenario were regressed against the output of the control scenario. A normalized index of the impact of the treatments on ET (evapotranspiration) and NPP was calculated using the following equation:


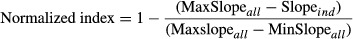


where MaxSlope_all_ is the maximum slope of the regression across all scenarios; Slope_ind_ is the slope for the regression for an individual scenario; and MinSlope_all_ is the minimum slope of the regression across all scenarios.

An index of zero means no impact of the treatment and an index of 1 means the largest treatment effect observed across all treatments (scenarios).

## Results

### The control scenario

**Figure 1:**
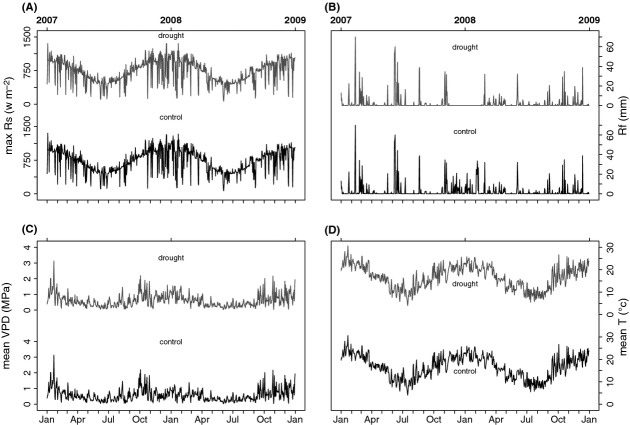
Meteorological input data (maximum solar radiation, rainfall, mean daily *D*, and mean daily air temperature) for control and drought simulations.

Solar radiation levels exhibited seasonal patterns, with larger values in the summer than winter (Fig. 1A). Consequently, mean *D* and air temperatures showed similar seasonal trends (Fig. 1C, D). Rainfall was approximately evenly distributed across the 2 years, although there was more rainfall in the first year (1074.4 mm) than the second year (839 mm; Fig. 1B) and slightly more rain in summer than winter, especially in the second year.

**Figure 2:**
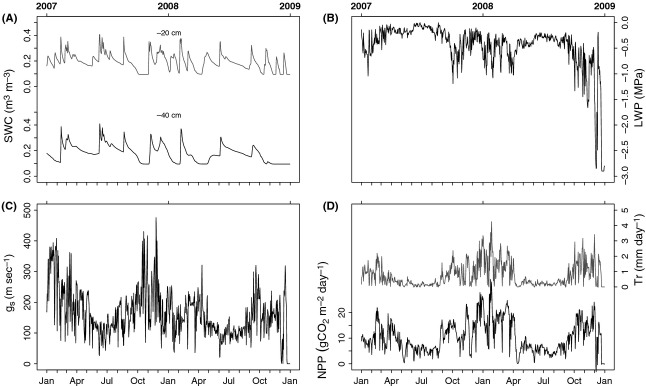
Soil water content at 20 and 40 cm depth, leaf water potential, daily average stomatal conductance, and NPP and transpiration rates for the simulation under control conditions. Summer maxima and winter minima in stomatal conductance, NPP, and transpiration rate are apparent in these model outputs.

Soil water content at 20 and 40 cm depth fluctuated between 10 and 40% across the 2-year simulation period in response to individual rain events (Fig. 2A). The shallower depth was more responsive to smaller rainfall events than the deeper profile, which required larger events for water to reach 40 cm. Because of relatively consistent rainfall across the first 20 months of the simulation, leaf water potential in the control simulation remained consistently high, ranging from −1.5 MPa to −0.2 MPa across the first 20 months. Leaf water potential was closer to zero in winter and declined in each summer period, typically to −1 MPa in the first two summers when temperatures and vapor pressure deficits were relatively large (Fig. 2B). In the final summer, leaf water potentials declined significantly because of reduced rain in the last 2 months of the second year of the simulation.

Mean daily *g*_s_ fluctuated between 100 and 400 mmol m^−2^ sec^−1^ across the 2-year simulation period, with larger values observed in the summer than the winter (Fig. 2C). Seasonal changes in *g*_s_ were reflected in seasonal changes in transpiration and NPP (Fig. 2D). Daily transpiration varied between 0.1 mm day^−1^ and 4 mm day^−1^ across almost all of the 2-year simulation, but then declined to zero at the end of the second year following a period of low rainfall during the final summer (December year 2). The same patterns in NPP were observed as for transpiration, with peak NPP observed in the summers and minima in winter.

### Impacts of increased temperature and increased *D* on soil water content, *g*_s_, leaf water potential, NPP, and ET

**Figure 3:**
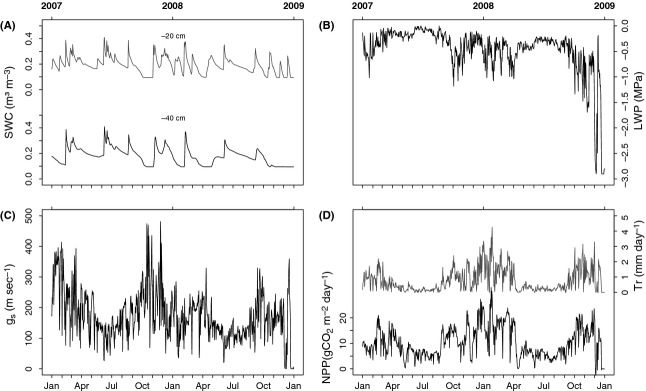
Soil water content at 20 and 40 cm depth, leaf water potential, daily average stomatal conductance, and NPP and transpiration rates for the +2°C temperature simulation. A comparison of Figure 2 with Figure 3 reveals very little difference in model outputs because the 2°C increase in temperature has little impact on forest function.

When temperature alone was increased by 2°C across the 2-year simulation period, patterns of soil moisture content (θ), leaf water potential, *g*_s_, transpiration, and NPP (Fig. 3A–D) were indistinguishable to those observed in the control scenario. Seasonal maxima in *g*_s_, transpiration, and NPP occurred in the summer, minima occurred in the winter, and the range in values across seasons was not affected by an increase in temperature. There were, however, very small changes in annual transpiration rate (increased by 1% in both years; Table 3) and NPP (declined by 1.3% and 1.7% in years 1 and 2, respectively), which resulted in one additional day (14 compared to 13) when NPP was zero in the +2°C scenario compared with the control scenario (Table 3).

**Table 3:** A summary of the impacts of the various modeling scenarios on the number of days for which NPP = 0 in years 1 and 2 and the cumulative NPP and transpiration (T) for years 1 and 2

	Number of days NPP = 0			Annual NPP (gCO_2_ m^−2^ year^−1^)					Annual *T* (mm)						
Year 1	Year 2	Year 1	Year 2	Year 1	Year 2
Control	0	13	3717.7	3973.7	242.0	313.2
+2°C	0	14	3667.0	3905.3	244.5	316.2
+5°C	0	13	3484.3	3723.6	245.0	322.1
+1 kPa *D*	0	197	3593.3	1442.6	367.3	169.0
+1 kPa *D* + 2°C	0	175	2802	1375	356.0	162.7
+2.5 kPa *D*	3	202	3467.0	1361.1	352.7	109.1
+2.5 kPa + 5°C	153	284	1525.5	481.2	303.6	111.4

*T*, transpiration rate.

**Figure 4:**
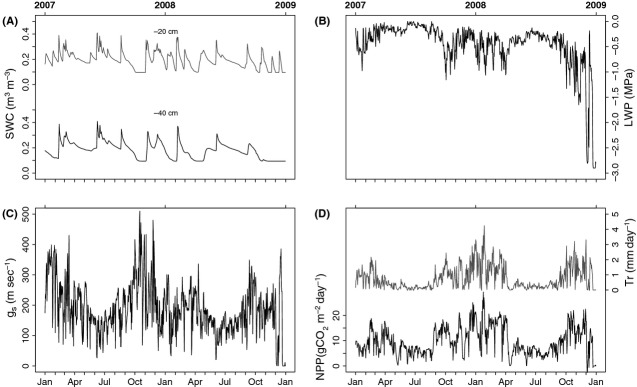
Soil water content at 20 and 40 cm depth, leaf water potential, daily average stomatal conductance, and NPP and transpiration rates for the +5°C temperature simulation. A comparison of Figure 3 with Figure 4 reveals only minor differences in forest function, for example, slightly larger maximum rates of stomatal conductance in Figure 4.

When temperature was increased by +5°C, the same seasonal pattern of change in θ, *g*_s_, leaf water potential, transpiration, and NPP was observed as for the control scenario (Fig. 4), with similar ranges in all variables observed. However, the annual sum of NPP was 6.3% lower in the +5°C scenario compared to the control, whereas transpiration was increased by 1.2% and 2.8% in years 1 and 2, respectively (Table 3).

**Figure 5:**
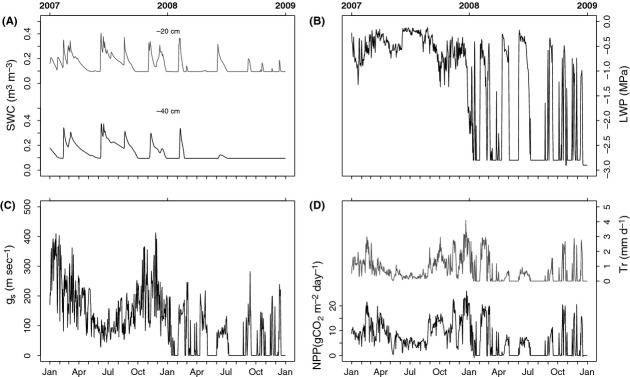
Soil water content at 20 and 40 cm depth, leaf water potential, daily average stomatal conductance, and NPP and transpiration rates for the +1 kPa increase in *D* simulation. This small increase in *D* has had large negative impacts on leaf water potential, stomatal conductance, NPP, and transpiration compared to the control or increased temperature scenarios.

**Figure 6:**
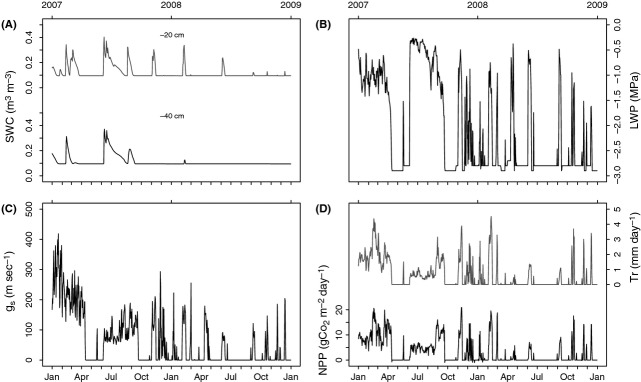
Soil water content at 20 and 40 cm depth, leaf water potential, daily average stomatal conductance, and NPP and transpiration rates for the +2.5 kPa increase in *D* simulation. Further increase in *D* has increased the magnitude and frequency of the negative impact of a small increase in *D* observed in Figure 5.

When vapor pressure deficit (*D*) was increased by 1 kPa across both years, new patterns in θ, leaf water potential, *g*_s_, and NPP and transpiration were observed (Fig. 5A–D). Soil water content at both 20 and 40 cm depth was reduced to 10% for extended periods in the latter half of year 1 and for extended periods throughout year 2. Consequently, leaf water potential declined to low levels (−3 MPa) for much of the second year of the simulation, which resulted in reduction in *g*_s_, NPP, and transpiration to zero for extended periods (Fig. 5B–D). Because of these responses, annual NPP and transpiration were significantly reduced (63.7% and 46%, respectively, in year 2) compared to the control scenario (Table 3). These patterns and impacts were exacerbated when *D* was increased by 2.5 kPa (Fig. 6). When θ and leaf water potentials were minimal, the number of days when NPP was zero was large (cf. Table 3 and Fig. 6A). The percentage decline in annual NPP and transpiration in year 2 increased from 63.7% and 46% (for +1 kPa increase in *D*) to 65.7% and 65.1%, respectively (for a 2.5 kPa increase in *D*; Table 3). The response of transpiration in year 1 to increased *D* (of 1 kPa or 2.5 kPa) differed from that of year 2. Thus, annual transpiration increased in year 1 compared to the control scenario (but transpiration decreased in year 2).

**Figure 7:**
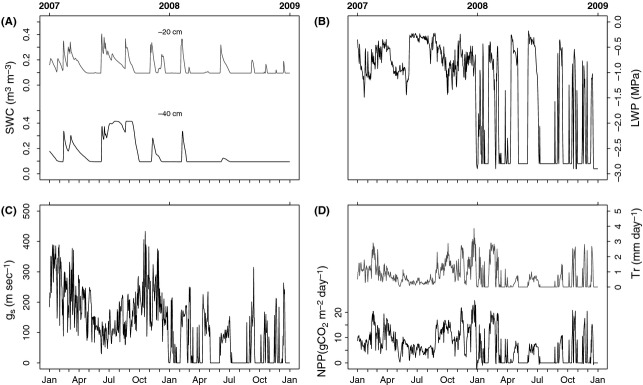
Soil water content at 20 and 40 cm depth, leaf water potential, daily average stomatal conductance, and NPP and transpiration rates for the +1°C plus 2.5 kPa increase in *D* simulation. A comparison of Figure 6 with Figure 7 reveals that the addition of a small increase in *T* to the +2.5 kPa *D* treatment did not cause major changes in forest function.

**Figure 8:**
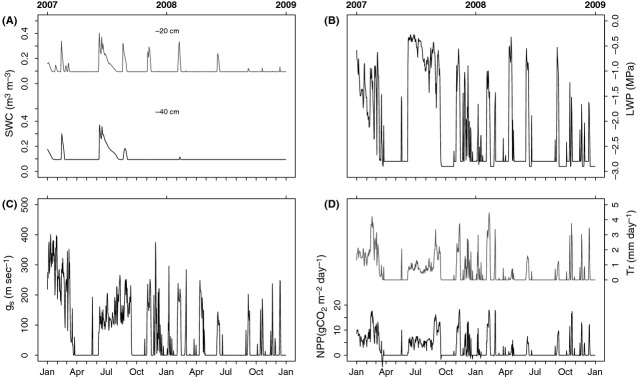
Soil water content at 20 and 40 cm depth, leaf water potential, daily average stomatal conductance, and NPP and transpiration rates for the +2.5°C plus 5 kPa increase in *D* simulation. A comparison of Figure 7 with Figure 8 reveals that the addition of a larger increase in *T* to the +2.5 kPa *D* resulted in reductions in stomatal conductance, leaf water potential, transpiration, and NPP.

When air temperature was increased by 2°C in combination with a 1 kPa increase in *D*, the pattern of responses for θ, leaf water potential, *g*_s_, and NPP and transpiration was similar to those observed when *D* was increased alone (compare Fig. 7A–D with Figs. 5, 6). The decline in annual NPP and transpiration in year 2 (65.4% and 48%, respectively) was slightly larger than that observed when increased *D* was applied alone (Table 3). When *D* was increased by 2.5 kPa in combination with an increased temperature of 5°C (Fig. 8), the number of days when NPP was zero increased in both years of the simulation (153 and 184 days for years 1 and 2, respectively; Table 3) and was the largest of all simulations that did not include a drought. Consequently, annual NPP was reduced by 59% and 87.9% in years 1 and 2, respectively, whereas transpiration increased in the first year by 25% but declined in the second year by 64.4% (Table 3).

### Impacts of drought interacting with increased temperature **or** increased *D* on soil water content, *g*_s_, leaf water potential, NPP, and transpiration

Drought was applied as a 3-month absence of rain during the summer spanning years 1 and 2 (Fig. 1B). Because of the drought, annual rainfall in year 1 was 930.4 mm (1074.4 mm without drought) and in year 2 rainfall was 574.2 mm (839 mm without drought).

**Figure 9:**
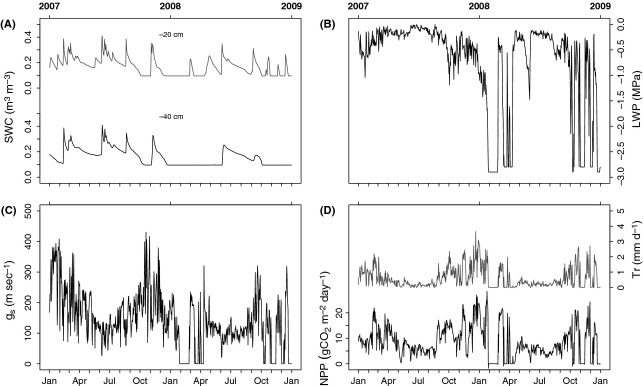
Soil water content at 20 and 40 cm depth, leaf water potential, daily average stomatal conductance, and NPP and transpiration rates for the drought simulation. The 3-month drought had a major effect on summer-time leaf water potential and stomatal conductance.

Drought resulted in the decline in θ to minimal values at both 20 and 40 cm depths for extended periods, both during and following the drought (Fig. 9A). Consequently, leaf water potential declined to minimal values (−3 MPa) during the drought and during the final summer toward the end of the second year (December) (Fig. 9B). This resulted in closed stomata (*g*_s_ = 0) toward the end of the drought and during the final summer of the second year. The number of days when NPP was zero increased from 13 (control) to 88 by the imposition of the drought, and annual NPP and transpiration declined in the second year of the simulation by 33.9% and 41.2%, respectively (Table 4), compared to control values.

**Table 4:** Drought interactions with *D* and temperature and the response of NPP and transpiration across the 2-year simulation period

	Number of days when NPP = 0			Annual NPP					Annual *T*						
Year 1	Year 2	Year 1	Year 2	Year 1	Year 2
Control	0	13	3717.7	3973.7	242.0	313.2
Drought	0	88	3694.2	2627.9	251.3	184.3
Drought + 2°C	0	87	3648.0	2601.4	253.8	186.2
Drought + 5°C	0	83	3465.4	2514.0	254.4	190.5
Drought + 1 kPa *D*	11	235	3334.6	944.5	338.0	99.8
Drought + 1 kPa *D* + 2°C	11	240	3308.0	923.3	341.9	100.4
Drought + 1 kPa *D* + 5°C	12	242	3195.3	893.8	345.2	102.7
Drought + 2.5 kPa *D*	140	304	1935.9	335.8	331.4	62.1
Drought + 2.5 kPa *D* + 2°C	144	306	1896.6	326.7	337.5	63.1
Drought + 2.5 kPa *D* + 5°C	147	307	1830.1	311.2	347.1	65.4

*T*, transpiration rate.

When drought was applied with an increased temperature of 2°C, θ was minimal for extended periods during and following the drought (Fig. 10A–D). However, the number of days when NPP was zero did not increase relative to the drought treatment applied alone, and there was a minimal reduction (1%) in annual NPP for years 1 and 2 compared to the drought treatment alone (Table 4). Annual transpiration rate increased slightly (1%) for the drought plus increased temperature scenario compared to the drought applied alone (Table 4). A similar pattern was observed when drought was applied with a 5°C temperature increase. Annual NPP in years 1 and 2 declined by 6.2% and 4.3% relative to the drought treatment applied alone, whereas transpiration increased by 1.2% and 3.3% in years 1 and 2, respectively (Table 4).

**Figure:**
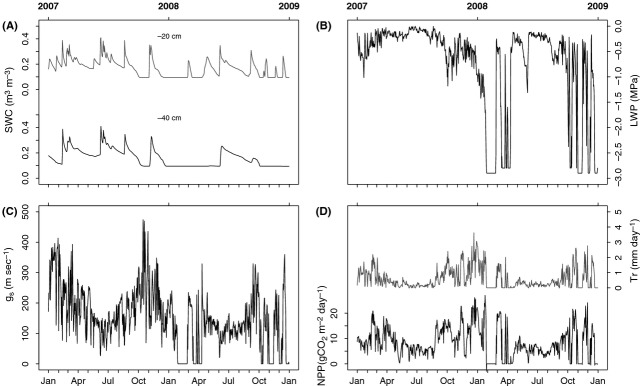
Soil water content at 20 and 40 cm depth, leaf water potential, daily average stomatal conductance, and NPP and transpiration rates for the drought +2°C temperature simulation. A comparison of Figure 9 with Figure 10 shows that a small increase in temperature had little impact on forest function compared to the impact of the drought treatment.

**Figure 11:**
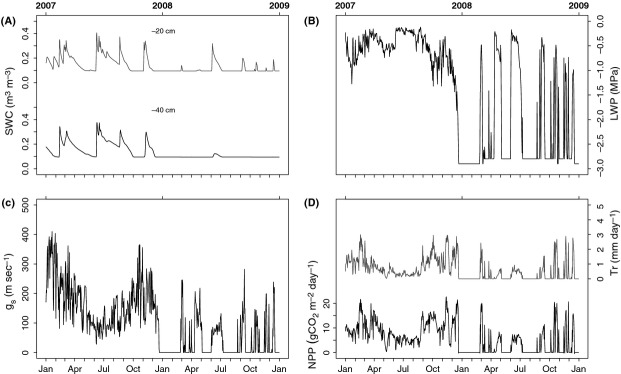
Soil water content at 20 and 40 cm depth, leaf water potential, daily average stomatal conductance, and NPP and transpiration rates for the drought +1 kPa increase in *D* simulation. A comparison of Figure 9 with Figure 1 shows that a small increase in *D* applied with the drought had a large negative impact on forest function.

When drought was applied simultaneously with increased *D* (1 kPa), there were extended periods in year 2 when θ and leaf water potential were minimal (−3.0 MPa; Fig. 11). Consequently, *g*_s_, NPP, and transpiration were zero for extended periods in year 2 (Fig. 11C, D). In years 1 and 2, annual NPP declined by 9.7% and 64%, respectively, whereas transpiration was larger in the first year (3.5%) but smaller in the second year (45.8% decline) compared to the drought treatment applied alone (Table 4). When drought was applied simultaneously with a larger increase in *D* (2.5 kPa), the number of days when NPP was zero increased substantially in both years of the simulation (to 140 and 304 days for years 1 and 2, respectively). Consequently, the decline in NPP (47% and 87% for years 1 and 2, respectively) was much larger for drought plus 2.5 kPa increase in *D* compared to drought plus 1.0 kPa increase in *D* (Table 4). In contrast, annual transpiration increased in the first year but showed a large decline (66%) compared to the impact of drought alone (33% lower than the drought plus 1.0 kPa increase in *D* scenario; Table 4).

### Impacts of drought interacting with increased temperature **and** increased *D* on soil water content, *g*_s_, leaf water potential, NPP, and transpiration

**Figure 12:**
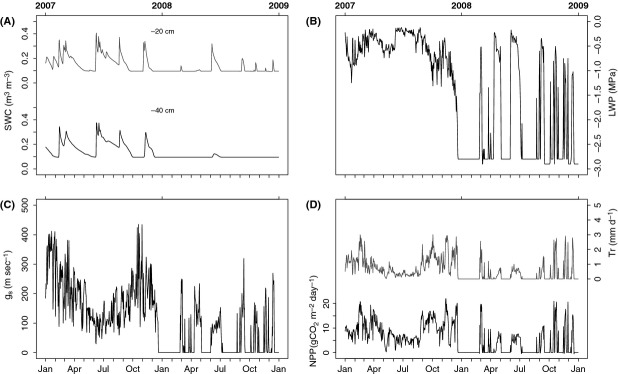
Soil water content at 20 and 40 cm depth, leaf water potential, daily average stomatal conductance, and NPP and transpiration rates for the drought +2°C plus 1 kPa increase in *D* simulation. A comparison of Figure 1 with Figure 2 shows that a small increase in temperature applied with the drought had minimal impact on forest function, compared to drought applied alone.

**Figure 13:**
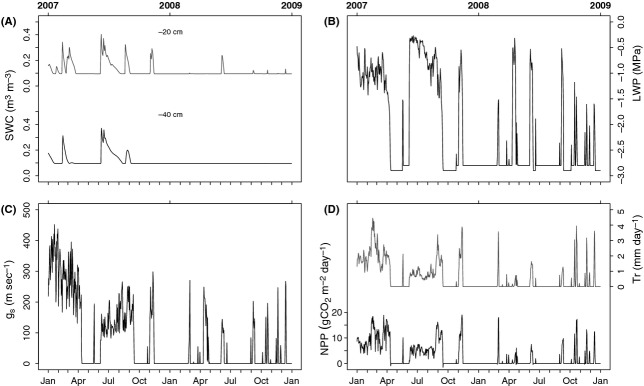
Soil water content at 20 and 40 cm depth, leaf water potential, daily average stomatal conductance and NPP and transpiration rates for the drought +5°C plus 2.5 kPa increase in *D* simulation. Large increases in temperature and vapor pressure deficit along with a summer drought reduced leaf water potential, stomatal conductance, transpiration rate, and NPP substantially.

When drought was applied with increased temperature and increased *D*, there were extensive periods beginning December of the first year when θ and leaf water potential (−3.0 MPa) were minimal (Figs. 12, 13). Consequently, the number of days when NPP was zero increased compared to all other scenarios (Table 4). The number of days when NPP was zero increased only slightly when temperature was added in combination with drought and increased *D* (drought plus 1 kPa or 2.5 kPa). Thus, drought plus 1 kPa increase in *D* and 2°C or 5°C increase in temperature resulted in a larger number of days when NPP was zero (from 246 days without the temperature treatment to 251 or 254 days, respectively). Similarly, drought plus 2.5 kPa with an increase of 2°C or 5°C increased the number of days when NPP was zero from 444 days to 450 or 454 days, respectively, and clearly indicating that the additional stress of increased temperature had little impact on the loss of NPP across the 2 years.

### An index of impacts of the 15 scenarios on NPP and transpiration

**Figure 14:**
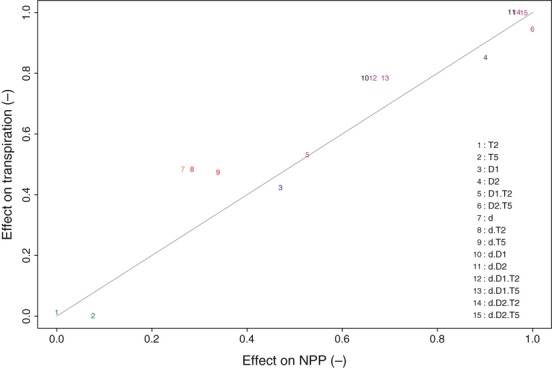
Normalized changes in NPP and transpiration resulting from increase in temperature, drought, VPD, applied as single factors or in combination. d, drought; *D*, VPD;* T*, temperature. It is clear that treatments with increased *T* alone had the smallest impact on transpiration and NPP, whereas increased *D* alone had significant negative impacts on both fluxes. The largest negative impacts on water and C fluxes occurred when drought and large increases in vapor pressure deficit were imposed. An increase in *T* had little impact to the influence of drought plus large VPDs.

The normalized impacts of the 15 scenarios on annual NPP and transpiration, expressed relative to the control scenario reveal several key insights (Fig. 14). Temperature treatments (+2°C or +5°C) had little impact on either NPP or transpiration as points 1 and 2 lie close to the origin. Points 3 and 4 (increased *D* of 1 or 2.5 kPa) increased the magnitude of the decline in NPP and transpiration substantially, whereas increased temperature with increased *D* (points 5 and 6) caused only a small change in the decline in NPP and transpiration relative to increased *D* alone (points 3 and 4).

Drought had a large negative impact on both NPP and transpiration (point 7). However, the interaction of temperature with drought (points 8 and 9) had little impact on transpiration and only a modest impact on NPP (Fig. 14). In contrast, the interaction of drought with increased *D* (points 10 and 11) had a larger negative impact on NPP and transpiration than the interaction of drought with increased temperature. Similarly, the interaction of temperature with drought and increased *D* (compare point 10 with points 12 and 13 or compare point 11 with points 14 and 15) also shows that temperature had minimal impact on the response of NPP or transpiration.

## Discussion

Our results show that drought plus increased *D* had a much larger negative impact on transpiration and NPP than drought plus increased temperature. The number of days when NPP was zero increased from 88 (drought alone) to 246 (drought plus 1 kPa increase in *D*) or 444 days (drought plus 2.5 kPa increase in *D*; Fig. 4). In contrast, drought plus a 2°C or 5°C increase in temperature did not cause an increase in the number of days when NPP was zero. Although tree health is difficult to quantify, we argue that a sustained decline in NPP is indicative of a loss of tree health. Carbon fixation is central to tree growth and even in the absence of growth, the maintenance of trees through time in a landscape requires all C losses (respiratory, exudation, mortality, and herbivory) to be compensated for by C uptake equal to the sum of all losses. Consequently, a sustained (many consecutive days) decline in NPP at a time when NPP would not normally be low is indicative of a decline in health. When C losses (e.g., through respiration or exudation) exceed C gain for a sufficient time, mortality must result as trees do not have unlimited reserves of stored carbon.

At a constant absolute atmospheric water content, when temperature is 30°C, an increase in temperature to only 35°C results in an increase in *D* of >1 kPa. Drought is associated with both increased temperatures (because more energy is lost as sensible heat rather than latent energy and cloud cover is reduced) and increased *D* (because of the increase in temperature and decreased evapotranspiration). Thus, the use of increased *D* of 1 kPa and 2.5 kPa in these simulations is reasonable. Similarly, increased air temperatures associated with drought can be in the range 2 to 10°C and therefore our choice of +2°C and +5°C increase appears reasonable.

Our application of the SPA model allows “what-if” questions (e.g., “what happens to NPP if *D* increases all year”) to be addressed at scales that are not amendable to experimental manipulation. The SPA model applied in this work is a detailed mechanistic model describing the processes of water uptake, transpiration, photosynthesis, and changes in stomatal conductance and soil water content. It has been successfully applied to a range of diverse ecosystems including Australian tropical savannas (Whitley et al. 34), temperate Australian woodlands (Macinnis-Ng et al. 20), Amazonian forests (Fisher et al. 13), and Arctic tundra (Williams et al. 37), and has been applied to the Australian Cumberland Plain woodland represented herein. However, it is noteworthy that the SPA model is based on an isohydric assumption (sensu Tardieu and Simmoneau 31) such that leaf potential is not allowed to decline beyond a critical threshold during drought; rather, stomatal closure occurs during (atmospheric or soil) drought to limit the decline in leaf water potential. This appears as a reasonable assumption; first, because the dominant tree species at this site limit their decline in leaf water potential during drought (Zeppel et al. 40); and second, the site used herein is a mesic temperate site, whereas anisohydric behavior is more commonly observed in more arid environments (McDowell et al. 23). The aim of this work was *not* to compare the behavior of anisohydric species with isohydric species; *nor* was the aim to compare the hydraulic failure versus C starvation hypothesis (McDowell et al. 23), recognizing that there may be a continuum of importance between the two and that they are interrelated (McDowell et al. 24); rather it was to compare the relative importance of increased *D* and temperature as determinants of changes in canopy processes that may cause forest dieback. However, as noted in McDowell et al. (23) both anisohydric and isohydric species may encounter hydraulic failure and C starvation if droughts are severe and persistent.

Our results highlight that increased mortality associated with climate change-type droughts is caused more by the increased *D* (associated with either drought or climate warming or both) than an increase in temperature alone (which is occurring during global warming). We note that in the field, increased temperature alone (i.e., without increased *D*) is rarely observed during droughts because of a decrease in partitioning of energy fluxes to latent energy fluxes. Our results are consistent with recent analyses considering *D* (Weiss et al. 32; Williams et al. 39), while additionally disaggregating the effect of *D* from that of temperature on tree physiological function. These result provide new insights related to the considerable evidence that widespread forest dieback (partial crown mortality) and die-off (extensive whole-plant mortality) has been experienced across multiple regions of the globe (Allen et al. 3): global change-type droughts (sensu Breshears et al. 6) and associated forest (including woodlands and savannas) has been observed in Africa, Australia, Europe, north, central, and south America, and Asia (for reviews see Allen et al. 3; McDowell et al. 24; Kumagai and Porporato 17).

Importantly, the severity and frequency of droughts and increased heat stress are predicted to increase during the 21st century, and these are the two most commonly implicated factors causing such widespread dieback and die-off (Horner et al. 14; Allen et al. 3; McDowell et al. 24), although the secondary influence of insect and pathogenic attack on *already weakened* trees is certainly important (Allen et al. 3). van Mantgem et al. (21) found a positive correlation between increased tree mortality and increased temperature. Similarly, Breshears et al. (6) suggested that recent droughts experienced by *Pinus edulis* in the United States were less severe than some previous droughts, but the impact on regional-scale mortality was larger in recent times and concluded that this was because of increased temperatures compared to earlier, more severe droughts. Adams et al. (1) subsequently showed experimentally that increased temperatures per se increased tree sensitivity to drought in a manipulation of temperature and water supply. However, as the authors note, their application of increased temperature also resulted in an increased maximum *D* of ~0.8 kPa, or a 50% increase over the controls (Adams et al. 1).

Dynamic global vegetation models (DGVMs) are process models that simulate population dynamics (including growth rate, mortality, and recruitment) of forests as a function of climate and forest composition. However, the mechanistic modeling of mortality remains in its infancy (McDowell et al. 24). Some models have a size, age, NPP, or temperature-driven mechanism for mortality (McDowell et al. 24). None, to the author's knowledge, distinguish between temperature per se and the associated changes in *D* that arise when temperature increases. Furthermore, drought is not only associated with decreased water availability but also increased temperature and *D* because of the change in partitioning of incoming solar radiation between latent and sensible heat fluxes.

Short-term drought applied alone had a minor impact on NPP in the first year, but a large impact in the second year of the simulation (33.9% and 41.2% reduction in NPP and transpiration, respectively). The drought reduced daily *g*_s_ by 50–100% (i.e., *g*_s_ was zero on some days) and the largest effect was experienced 1–2 months or 9–12 months (i.e., the following summer) after cessation of the drought. Such delayed effects (lag effects) of drought on canopy function have been observed in field studies (Allen et al. 3). The cause of the decline in *g*_s_ was the decline in leaf water potential, which was substantial toward the end of the drought period and in the following (non-droughted) summer. The cause of the decline in leaf water potential was the significant decline in soil moisture content at 20 and 40 cm depths during the drought period and many of the subsequent 9 months. Note that in this simulation, drought was not accompanied by increased temperature nor increased *D*.

Increased temperature of 2°C or 5°C applied alone had very little impact on canopy function (NPP and transpiration) or physiology (leaf water potential and *g*_s_; Fig. 14). Indeed, NPP declined by less than 2% (+2°C) or 6.3% (+5°C) across both years. Patterns in θ, *g*_s_, and leaf water potential across the 2 years were indistinguishable from the control scenario. Although both temperature increases caused NPP to decline slightly, transpiration increased slightly (1–3%) because of a small increase in average *g*_s_. As leaf temperature increases, photorespiratory losses of fixed carbon and mitochondrial respiration increase (Labate et al. 18), which may explain the small decrease in NPP despite the small increase in *g*_s_ (and hence transpiration). Small increases in transpiration coupled with small decreases in NPP would have caused increased water-use-efficiency (WUE). Although WUE is generally found to decline with increased temperature, this is mostly associated with the increased *D* that accompanies increased temperature in most experimental manipulations (Barton et al. 5). Leaf water potentials were lower when temperature was increased, reflecting the enhanced transpiration observed in some days. Importantly soil moisture content at neither depth was affected by either of the two temperature treatments. We conclude that neither the 2°C nor the 5°C increase in temperature caused NPP to decline significantly and therefore we conclude that tree health (defined here as long-term declines in NPP) was unaffected by temperature increases alone.

Increasing *D* alone had large negative impacts on NPP and transpiration in the second year (Table 3 and Fig. 14). A small increase in *D* of 1 kPa caused increased rates of transpiration in the early part of the first year of the simulation because of increased soil evaporation and transpiration when soil moisture supplies were sufficient to support it. However, this increase was unsustainable and during the latter part of the first year and throughout the second year of the simulation, large reductions in θ, particularly at 40 cm depth but also at 20 cm depth, occurred with concomitant declines in leaf water potential to –3.0 MPa and hence reduced stomatal opening for extended periods (7 weeks in July and August of the second year of the simulation). These patterns of changing θ, leaf water potential, and *g*_s_ resulted in a small decline (3.3%) in annual NPP in year 1, but a substantial decline during year 2 (63.7% decline). We suggest that this decline in NPP for much of the second year of the simulation will result in increased rates of mortality. Increased mortality may occur because of depletions of carbon stores within the tree because of isohydric behavior of stomata causing stomatal closure in the absence of xylem embolism, or through stomatal closure arising from xylem embolism in anisohydric species (Allen et al. 3). As the SPA model is essentially a model based upon isohydric behavior of *g*_s_, we cannot distinguish between the two possibilities from these results. The cause of the decline in *g*_s_ and NPP to zero in the second year was the consequence of larger transpiration in the first year, which depleted soil moisture stores at both 20 and 40 cm depths.

Increasing *D* by 2.5 kPa alone exacerbated the problems observed with an increased *D* of 1 kPa. Soil moisture content was minimal at both depths within 4–5 months of increased *D* treatment, and θ at 40 cm depth remained minimal for almost the entire final 15 months of the simulation (Fig. 6C). Stomatal conductance and NPP declined to zero earlier such that zero values were observed from May of the first year. The longest period of zero NPP increased from 7 weeks to 9 weeks due to increased *D*. In the second year of the simulation, cumulative annual NPP was reduced by 65.7% and the number of days when NPP was zero increased from 175 to 205 days (Table 3). We conclude that canopy health was substantially decreased by the imposition of an increase in *D* of 2.5 kPa, which would allow increased attack by pathogens and insects and result in significantly larger rates of mortality compared to the control scenario.

When *D* increased by 1 kPa and temperature increased by 2°C, NPP declined in the first year by 25% compared to the control, but transpiration *increased* by 47%. In the second year, NPP declined by 65% and transpiration decreased by 48% (Table 3). Such declines were a function of increased photorespiratory losses and mitochondrial respiratory losses of C and reduced fixation of C arising from prolonged periods of stomatal closure in the second year. However, the decline in NPP in the second year was barely larger (65% compared to 63.7%) than that observed during the second year of the increased *D* (1 kPa) scenario. We again conclude that increased temperature per se has little impact on the behavior and function of the forest canopy, relative to the impact of increased *D*.

When drought was applied in combination with increased temperature or increased *D*, or in combination with both stress factors, the impact on NPP (and transpiration) was larger than when drought was not applied. We specifically imposed a short-term (but significant in terms of a total absence of rainfall for the summer) drought so that we did not push the physiology of the canopy so far that there was no capacity for the canopy to respond additionally to increased temperature and/or *D*.

A major finding of this study was that drought plus increased temperatures (of 2°C or 5°C) caused only minor declines (<6.5%) in NPP and only minor increases in transpiration compared to the drought treatment alone (Table 4 and Fig. 14). Furthermore, there was no increase in the number of days when NPP was zero when drought occurred with increased temperature, thus if mortality is associated with extended periods when NPP is zero, we would not expect any increase in mortality when temperature was increased with drought. This result supports the hypothesis that temperature per se is not the most significant factor associated with drought that causes increased dieback and die-off (mortality).

The second and perhaps more important key finding is that drought plus increased *D* had a much larger negative impact on NPP and transpiration than drought plus increased temperature. The number of days when NPP was zero increased from 88 (drought alone) to 246 (drought plus 1 kPa increase in *D*) or 344 days (drought plus 2.5 kPa increase in *D*). Thus, annual NPP declined by 64% in comparison to the drought treatment alone. In contrast, drought plus a 2°C or 5°C increase in temperature increased the number of days when NPP was zero to only 87 and 83 days, respectively, but NPP declined by only 1% relative to the drought treatment. This very large effect of increased *D* in combination with drought arose from the combination of increased soil evaporation, reduced rainfall, and more rapid depletion of soil moisture following each rain event, which resulted in extended periods of stomatal closure and hence reduced NPP. This conclusion supports the recent analyses of climatic limits on foliar growth during drought in the southwest United States. (Weiss et al. 33). These authors conclude that *D* constraints were limiting to growth, particularly during drought. It is pertinent to note that decadal averages of *D* (1983–2008) can show both increasing (1990–1999) and decreasing (1983–1989) trends (Matsoukas et al. 22), with cycles of solar radiation output possibly explaining these variations (Zhang et al. 41). However, irrespective of such large-scale and long-term fluctuations in *D*, drought at any given site is accompanied by increased temperature and concomitant increases in *D*. We conclude that mortality is caused more by the increased *D* associated with either drought than an increase in temperature alone, which can occur during global warming, but which is rarely observed during droughts in the field.

We note that recent field studies of mortality in conifers (Weiss et al. 32; Williams et al. 39) and our SPA results for eucalypt woodlands all highlight a critical role for *D* in causing substantial and long-term declines in tree vigor, and by implication, increased probability of mortality in two of the most widespread forest genera. As such there are clear implications globally. Notably, recent assessments have likely underestimated future mortality.

## Conflict of Interest

None declared.
